# Diffusion weighted imaging in musculoskeletal system: where are we now?

**DOI:** 10.1093/bjro/tzaf019

**Published:** 2025-07-22

**Authors:** Sonal Saran, Avneesh Chhabra, Rajesh Botchu

**Affiliations:** Department of Diagnostic and Interventional Radiology, AIIMS Rishikesh, Rishikesh, Uttarakhand, 249203, India; Department of Radiology, UT Southwestern Medical Center, Dallas, TX, 75390, United States; Department of Musculoskeletal Radiology, Royal Orthopedic Hospital, Birmingham , B31 2AP, United Kingdom

**Keywords:** diffusion-weighted imaging (DWI), musculoskeletal disorders, apparent diffusion coefficient (ADC), diffusion tensor imaging (DTI), tumour characterization

## Abstract

Diffusion-weighted imaging (DWI) is an advanced MRI technique that harnesses the movement of water molecules within tissues to assess and characterize a wide range of musculoskeletal disorders. By differentiating between isotropic and anisotropic diffusion, DWI provides critical insights into tissue integrity and pathology, proving instrumental in diagnosing conditions. Its sensitivity to changes in tissue microstructure is quantified through metrics like the apparent diffusion coefficient (ADC) and fractional anisotropy (FA). Advanced methodologies, including diffusion tensor imaging (DTI) and diffusion kurtosis imaging (DKI), further enhance DWI's ability to evaluate complex tissue architectures, offering vital information on muscle, ligament, tendon, and cartilage health. DWI also excels in the assessment of soft tissue tumours, infections, and joint pathologies, enabling accurate differentiation between benign and malignant lesions and facilitating early detection of conditions like osteomyelitis. Additionally, DWI plays a crucial role in monitoring treatment responses, with ADC changes correlating to tumour necrosis and recurrence. Despite its advantages, DWI faces limitations, such as technical artefacts and challenges in interpretation that can impact diagnostic accuracy. This review explores the diverse applications of DWI and DTI in musculoskeletal imaging, highlighting their potential to improve diagnostic precision and clinical outcomes while addressing ongoing challenges in the field.

## Background

Diffusion-weighted imaging (DWI) is a sequence in magnetic resonance imaging (MRI) that utilizes the movement of water molecules in living tissue to characterize various abnormalities. This method is particularly adept at detecting variations in tissue organization, providing valuable insights that complement traditional clinical MRI approaches. DWI capitalizes on the Brownian motion of water molecules within living tissues. In “unrestricted environments,” this motion is random (isotropic diffusion), but in vivo, the complex internal architecture of tissues—including cell membranes and macromolecules—impedes diffusion, resulting in “restricted diffusion” or “anisotropic diffusion.” Consequently, regions with “slow or restricted diffusion” manifest as “hyperintense” on DWI, while areas characterized by “rapid diffusion,” such as extracellular spaces or blood vessels, appear “hypointense.” This ability to differentiate between various tissues and lesions based on their diffusion characteristics significantly enhances conventional MRI, allowing radiologists to make more accurate and confident diagnoses.^1^

The well-structured nature of most musculoskeletal connective tissues makes them particularly amenable to DWI analysis. A deterioration in the structural integrity of these tissues is often associated with altered diffusion characteristics. Therefore, DWI emerges as an important imaging modality for assessing a range of musculoskeletal disorders and predicting their progression. Following its initial success in brain imaging, DWI has gained traction in musculoskeletal radiology, increasingly playing a pivotal role in diagnostic strategies and in evaluating the therapeutic response of bone and soft tissue tumours.^2–4^ In addition to delivering qualitative evaluations of tissues and lesions with excellent background suppression, DWI and its variations, such as “diffusion tensor imaging (DTI),” also provide quantitative metrics like the “apparent diffusion coefficient (ADC),” “fractional anisotropy (FA),” and mean diffusivity (MD), which further refine lesion characterization.^5^^,^^6^ As we discuss the utility of DWI within the musculoskeletal system, it is essential to recognize both the notable achievements and the persistent challenges. This article will review the latest research findings and clinical applications for DWI in musculoskeletal imaging.

## Technical consideration of diffusion-weighted imaging in the musculoskeletal system

### DWI acquisition

**Figure 1.**
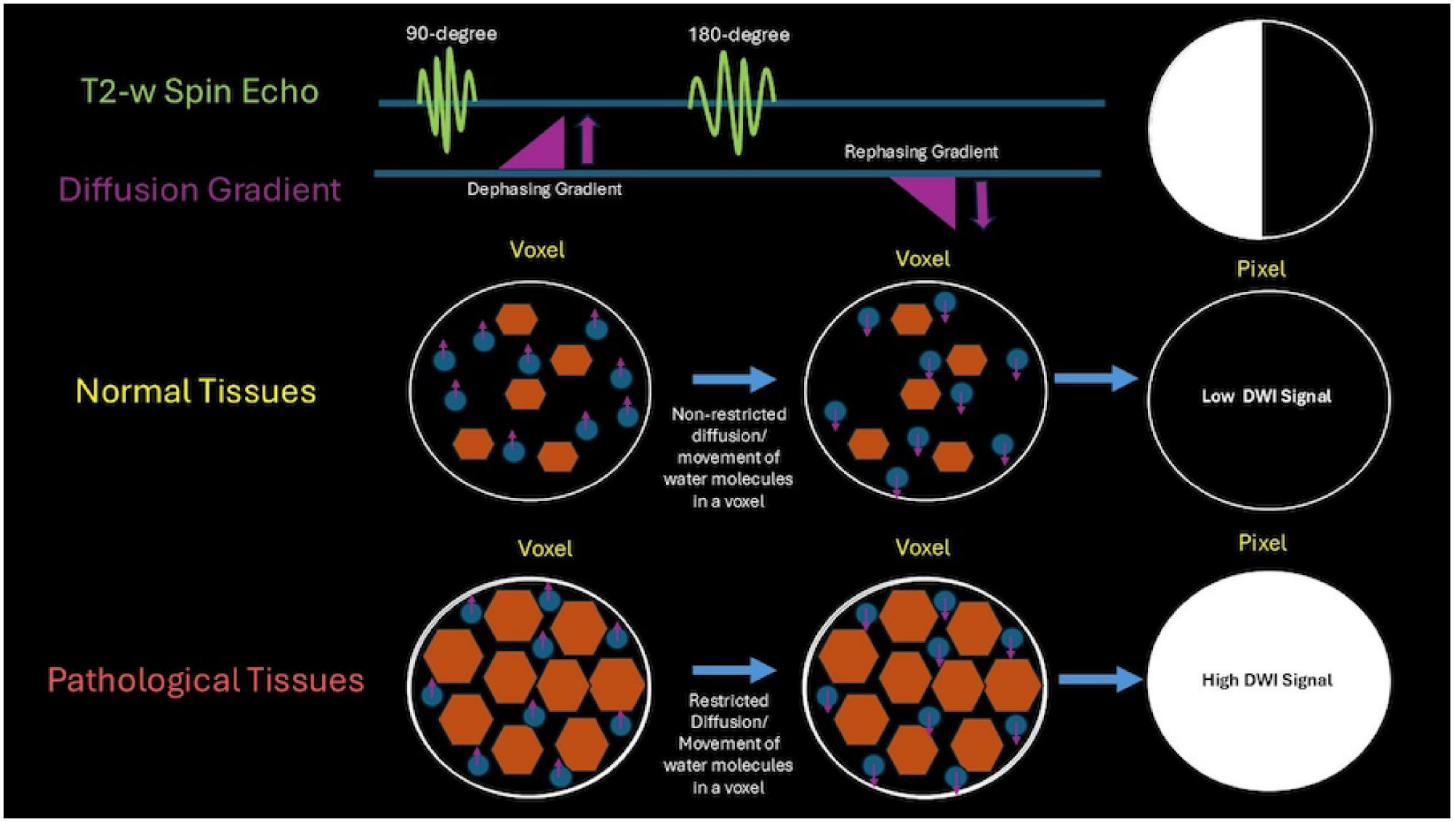
Applying 2 gradients in equal but opposite directions during a T2 sequence leads to a net signal loss from freely moving water molecules, while water that is restricted in motion retains a bright signal.

In DWI, magnetic field gradients play a crucial role in sensitizing MRI signals to the movement of protons within biological tissues. The process begins with a 90-degree radiofrequency (RF) pulse that excites protons in the tissue. Following this, a diffusion-sensitizing gradient is applied, causing protons in moving water molecules to experience varying magnetic field strengths depending on their spatial locations. This gradient induces a phase shift in the signals emitted by water molecules based on their diffusion behaviour. A time delay is introduced, during which the protons continue to diffuse. This delay allows for the measurement of how far the water molecules have moved. After this period, a second gradient is applied in the same direction as the first, aimed at rephasing the signals from water molecules that have not undergone significant movement. Water molecules that have diffused will not rephase correctly, leading to reduced signal intensity (Figure 1). The remaining signal is then collected, and this process is repeated with variations in gradient orientations and b-values, enabling the capture of diffusion information from various directions.

Higher b-values increase the sensitivity of DWI to diffusion but can also increase the levels of noise in the images. A b-value of 500-800 s/mm^2^ is crucial to minimize the influence of perfusion on the DWI signal, ensuring that the contrast primarily reflects water diffusion. By employing multiple b-values, clinicians can achieve a more precise assessment of diffusion characteristics. The acquired data are subsequently processed to generate DWI images and calculate the ADC, which aids in distinguishing between healthy and pathological tissues based on their diffusion properties. The ADC is calculated from the signal decay across multiple b-values, representing overall water diffusivity independent of direction.^7^^,^^8^ The region of interest (ROI) for ADC should be of moderate size (50-100 mm^2^) and placed in viable, non-necrotic tumour areas. Artefacts, oedema, and normal tissues should be avoided while placing the ROI. When reporting ADC values in musculoskeletal imaging, it is essential to specify the size/placement of the ROI and whether you are using minimum, mean, maximum, or ratio-based values, as each has different clinical implications. Comparing to reference tissue when possible (eg, “ADC ratio lesion/muscle”) reduces inter-scanner variability.

DWI can be implemented in 2 primary ways: single-shot and multi-shot acquisitions. Single-shot echo planar imaging (EPI) captures the entire image in one rapid acquisition, minimizing motion artefacts and making it suitable for patients who struggle to stay still, though it typically results in lower resolution and is more susceptible to distortion, especially at higher b-values. Conversely, multi-shot EPI acquires images over several cycles, allowing for higher spatial resolution and better handling of high b-values, but it requires complex phase correction to manage artefacts from motion between shots.^9–11^ This distinction is important as it influences the quality and reliability of the imaging results.

Selecting appropriate b-values is essential for achieving optimal image quality and diagnostic effectiveness. Typically, a combination of b-values is used: low b-values (eg, 0-100 s/mm^2^) provide baseline images but can be affected by T2 shine-through effects; intermediate b-values (eg, 400-600 s/mm^2^) offer a good balance by reducing perfusion influence while highlighting tissue microstructure; and high b-values (eg, 800-1000 s/mm^2^ or higher) enhance sensitivity to diffusion restriction, which is useful for identifying pathological changes such as tumours or fibrosis. The choice of b-values should be tailored to specific clinical needs, considering the anatomical region and the diagnostic question to optimize sensitivity and specificity.

### Other diffusion-based sequences

In the field of musculoskeletal imaging, advanced techniques like DTI and diffusion kurtosis imaging (DKI) have been explored by clinicians and researchers. These specialized methods offer detailed insights into the microstructural properties of tissues. Physically, DTI is based on the principle that water diffusion is anisotropic in structured tissues like muscles and tendons, meaning it varies with direction. The acquisition involves applying multiple diffusion gradients in at least 6 non-collinear directions, allowing for the calculation of diffusion tensors that describe the 3-dimensional diffusion profile. This process involves the use of EPI sequences to rapidly capture data and generate metrics like FA and MD, which reveal tissue structural integrity and orientation. MD is obtained by averaging the eigenvalues (λ_1_, λ_2_, λ_3_) of the diffusion tensor, reflecting the magnitude of isotropic diffusion. FA, ranging from 0 (isotropic) to 1 (anisotropic), quantifies directional preference by comparing eigenvalues (√[(λ_1_−λ_2_)^2^+(λ_2_−λ_3_)^2^+(λ_3_−λ_1_)^2^]/√2(λ_1_^2^+λ_2_^2^+λ_3_^2^)). By mapping these fibre orientations, DTI can help in identifying injuries, such as tears or strains, and in assessing changes in tissue composition due to various conditions.

DKI examines the non-Gaussian diffusion of water molecules. While DTI assumes diffusion follows a Gaussian distribution, DKI accounts for more complex diffusion behaviours, providing a richer dataset. DKI calculates mean kurtosis (MK), axial kurtosis (AK), and radial kurtosis (RK) to capture restricted diffusion caused by cellular membranes, organelles, and extracellular heterogeneity. These metrics are derived by applying higher b-values (typically >1000 s/mm^2^) and multi-directional gradients, fitting the signal decay to a kurtosis model. DKI provides enhanced sensitivity to microstructural complexity, particularly in regions with dense tissue organization (eg, brain gray matter, cartilage, or tumours), offering complementary insights beyond DTI. DKI is particularly useful in evaluating pathological conditions like inflammation or oedema, as it can detect variations in tissue density and organization.^6^^,^^12^

## Diffusion-weighted imaging of musculoskeletal tissues and their pathologies

Musculoskeletal tissue relaxation properties are heavily influenced by their composition, especially the presence of collagen. Collagen affects the behaviour of water molecules, leading to variations in signal intensity that depend on fibre orientation in anisotropic tissues such as cartilage and tendons. This interaction is crucial for understanding how these tissues function and respond to different conditions.^13^^,^^14^ This section will explore the role of diffusion-based sequences in evaluating various types of musculoskeletal tissues based on their specific composition. By leveraging this, clinicians can gain insights into the structural and functional aspects of tissues, helping to identify abnormalities and guide treatment decisions.

### Bone marrow

MRI of bone marrow presents unique challenges, particularly for DWI. The bone marrow consists of red and yellow marrow, which influence MRI signal and contrast within each voxel. The ADC of normal, healthy bone marrow is notably less than most of other soft tissues. Evaluating bone marrow with DWI can be done quantitatively by measuring ADC values or qualitatively by assessing diffusion-weighted image contrast in lesions. Red marrow and yellow marrow have different ADC values. Red marrow, due to its higher water content and vascularity, typically has a higher ADC (ranging from 0.902 to 1.34 × 10^−^³ mm^2^/s) than yellow marrow (ranging from 0.362 to 0.5 × 10^−^³ mm^2^/s).^15^^,^^16^

**Figure 2.**
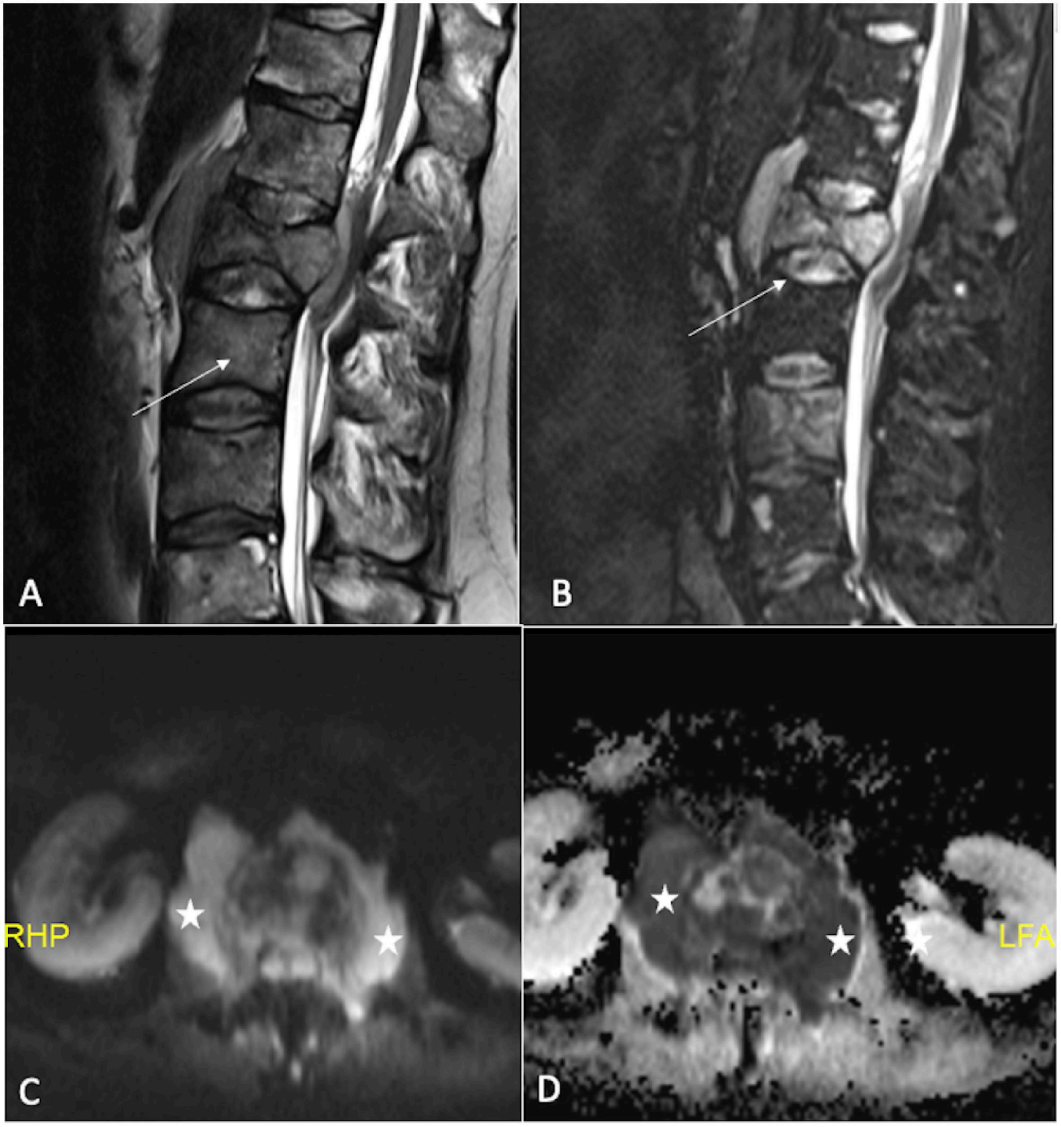
Bone lymphoma. A 45-year-old male showed multiple osteolytic lesions affecting several vertebral bodies, accompanied by compression collapse and associated soft tissue components (indicated by arrows in A and B). DWI shows hyperintense signal (marked by stars in C) in the soft tissues, with a corresponding marked ADC restriction (ADC = 0.6×10^−3^mm^2^/s), typically seen with round cell tumours (also marked by stars in D).

**Figure 3.**
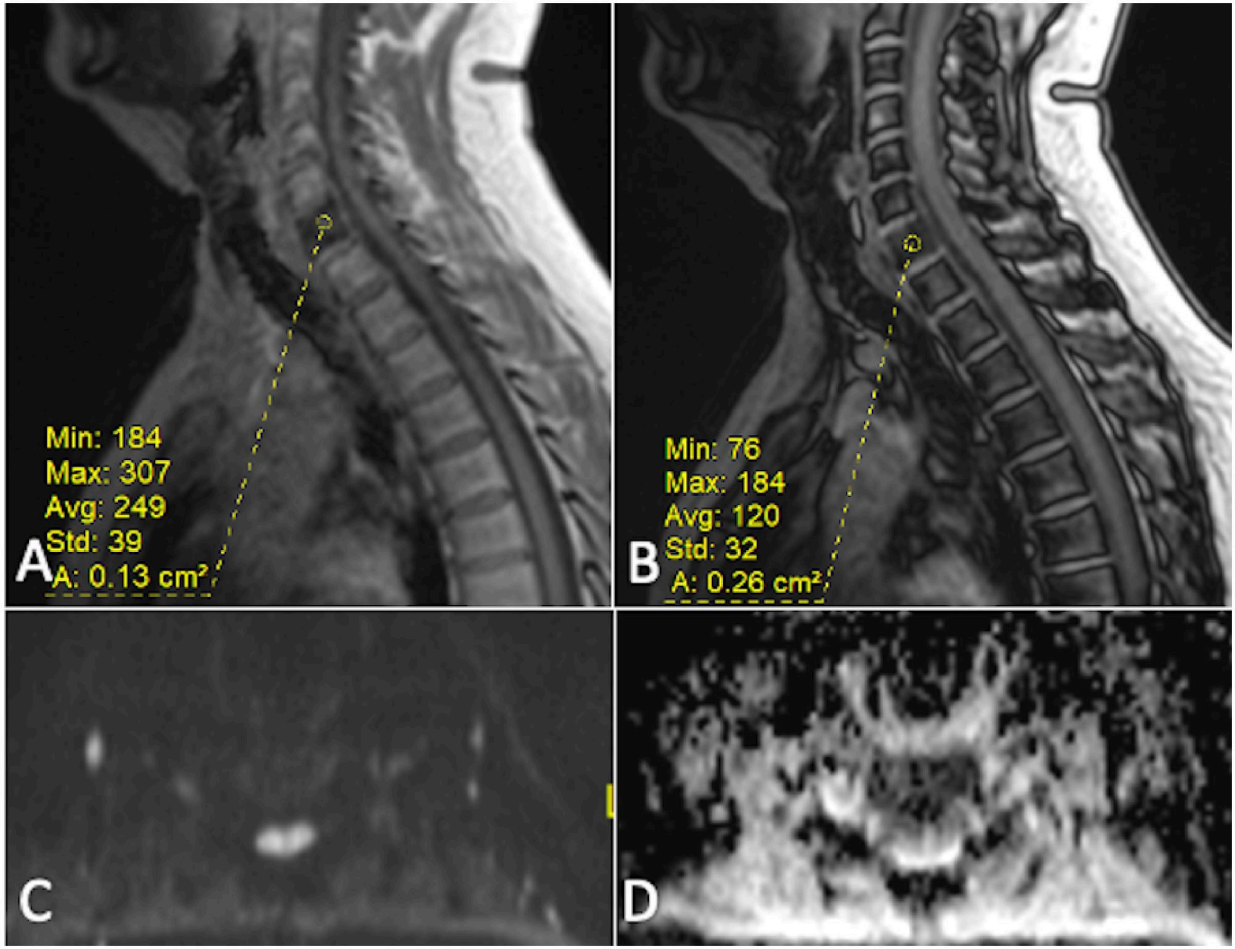
A focal area of red marrow hyperplasia in the C7 vertebra exhibits a 51.8% drop in signal on chemical shift imaging (A and B). This area shows no signs of diffusion restriction (C and D). More than 50% drop in signal intensity on out-of-phase chemical shift MRI images of the vertebral body, compared to in-phase images, indicates a benign lesion or normal bone marrow containing both fat and water, which cancel each other’s signals on the out-of-phase images.

DWI is particularly useful for diagnosing conditions such as osteoporotic (benign) vertebral fractures and malignant vertebral fractures resulting from metastatic tumours, multiple myeloma,^17^^,^^18^ or leukaemia^19^^,^^20^ (Figures 2 and 3). For instance, Wonglaksanapimon et al demonstrated the effectiveness of ADC (threshold of 0.89 × 10^−^³ mm^2^/s) in distinguishing benign from malignant causes of vertebral compression fractures, finding significant differences in ADC values.^21^ Dietrich et al observed that osteoporotic fractures had higher ADC values compared to malignant fractures.^15^ Additionally, Suh et al conducted a systematic review revealing pooled sensitivities of 89% and 92%, and specificities of 87% and 91% for differentiating benign and malignant vertebral lesions and compression fractures, respectively, noting that thinner DWI slices improved specificity.^22^ Furthermore, Cao et al found that vertebral metastases had significantly lower ADC compared to atypical hemangiomas, reinforcing its utility in distinguishing these conditions.^23^

### Ligaments, tendons and fibrocartilage

Ligaments, tendons, and fibrocartilage are connective tissues characterized by dense networks of collagen fibres. Their unique collagen arrangement and high water content provide distinctive imaging characteristics, especially for DWI. As these structures are highly ordered, they have been extensively evaluated using DTI. Among ligaments, the anterior cruciate ligament (ACL) is the most studied. Chen et al used DTI to evaluate the ACL in healthy adults, finding it effective for analysing ACL orientation and connectivity.^24^ Yang et al extended this by exploring DTI metrics for both native ACLs and ACL grafts, demonstrating its capability to visualize these structures.^25^ Van Dyck et al focused on DTI's reliability in assessing ACL grafts and showed that DTI could reliably visualize and quantitatively assess ACL grafts over time, determining their healing.^26^ In a longitudinal study, they observed significant decreases in FA in ACL grafts within the first year post-surgery, stabilizing thereafter, while diffusivity values returned to normal by 24 months. In contrast, the posterior cruciate ligament (PCL) showed no significant DTI changes.^27^ Liu et al investigated DTI's performance in assessing ACL injury severity and graft development within 6 months of surgery, concluding that DTI effectively quantified injury grades and graft maturation, with FA values showing higher diagnostic efficiency than ADC values.^28^

Wengler et al evaluated the feasibility and reliability of DTI for non-invasive assessment of the patellar tendon’s microstructure and microcirculation. They found strong test-retest reproducibility and interrater reliability, indicating DTI's clinical feasibility.^29^ Additionally, DTI is being applied to study other tendons, including the Achilles and rotator cuff tendons.^30–36^ These studies collectively underscore DTI's significance in identifying microstructural abnormalities not detectable with traditional radiologic methods. For fibrocartilage, some studies focus on the menisci. Shen et al examined porcine meniscus using 7.0 T MRI and found that diffusion properties varied by zone, suggesting DTI's usefulness in assessing meniscus integrity and visualizing collagen fibre architecture.^37^ Zhao et al used a high angular resolution diffusion imaging (HARDI) acquisition with both DTI and generalized q-sampling imaging (GQI) and provided a more accurate depiction of the complex 3D collagen network compared to DTI, which could aid in evaluating both normal and abnormal menisci in future studies.^38^

### Skeletal muscle

Water diffusion within muscle tissue is influenced by its structural complexity. Diffusion along the myofibers is relatively unrestricted, while perpendicular diffusion is limited by the sarcolemma and surrounding connective tissues within and around fascicles. DTI of muscle provides valuable insights into muscle architecture by enabling muscle fibre tracking, visualization of fibre insertion and orientation, and assessment of properties such as sarcolemma permeability. This information is crucial for identifying early markers of disease progression, particularly in idiopathic inflammatory myopathies (IIM) and various neuromuscular disorders, with DTI analysis revealing significantly higher average ADC values in IIM cases (2.07 ± 0.45) compared to controls (1.76 ± 0.26).^39^^,^^40^

Qi et al evaluated IIM patients using DWI of thigh muscles, noting that inflamed muscles exhibited elevated ADC values compared to unaffected ones.^41^ Faruch et al, in their study, found a positive correlation between ADC values and the grading of muscle oedema in IIM patients. DWI can detect muscle oedema before it is visible on the STIR sequence.^42^ Ai et al, in their study, found higher average ADC, FA, and eigenvalues in the muscles of IIM patients as compared to controls.^43^ Similar results were achieved by other authors.^44^^,^^45^ No studies have yet explored the role of DWI or DTI in characterization of IIM and assessing treatment monitoring in cases of IIM.

### Articular cartilage

Articular cartilage, a thin layer of connective tissue covering bone surfaces at joints, is primarily composed of water (about 80%) and a solid matrix consisting of aggrecan and a collagen framework. The integrity of the collagen network is crucial and is a key target for diffusion-based imaging sequences.^46^^,^^47^ Both DWI and DTI are valuable for assessing the collagen network within cartilage, providing insights into tissue integrity and the effectiveness of cartilage repair processes. DTI exploits the differential effects of cartilage matrix components on water diffusion. The highly organized collagen network promotes directional water movement along fibre orientations, producing measurable anisotropy quantified by FA. In contrast, proteoglycans exhibit no structural orientation, isotropically restricting water diffusion in all directions. Consequently, proteoglycan content primarily influences mean diffusivity (MD) without contributing to anisotropy. Welsch et al investigated the role of DWI in differentiating between cartilage repair tissue and healthy cartilage, concluding that DWI effectively distinguished the 2.^48^ Xu et al assessed the utility of T2-mapping and DWI for diagnosing early knee cartilage damage, finding that ADC values were elevated in early cartilage injury compared to healthy cartilage.^49^ In an ex vivo study, Ferizi et al examined the role of DTI in detecting articular cartilage injury, demonstrating that DTI is effective in identifying early changes following mechanical injury, with these changes correlating with alterations in biomechanics and histology.^50^

However, diffusion imaging cannot quantitatively assess the absolute PG or collagen content. A key limitation of this technique is its technical complexity, as conventional diffusion sequences lack sufficient image resolution to reliably measure diffusion properties in cartilage.

### Growth plate

The physes are cartilaginous plates located at the ends of immature bones, crucial for longitudinal bone growth. Along with the adjacent bone metaphysis, they exhibit a columnar architecture where growth is driven by the proliferation and enlargement of chondrocytes within each column. Water diffusion is facilitated along these cartilage columns in the physis and the newly formed bone within the metaphysis, primarily following the bone’s longitudinal axis, which aligns with the direction of growth.^51^^,^^52^

During skeletal growth, various skeletal disorders can arise from abnormal growth due to altered physeal activity. These can be generalized, such as in growth hormone deficiency, or isolated, as seen post-trauma. Partial physeal damage, often resulting from trauma or infection, can lead to angular deformities. Accurately assessing physeal activity provides valuable insights for diagnosis, treatment planning, monitoring therapeutic response, and predicting outcomes. DTI shows promise as a biomarker for evaluating physeal activity with high reproducibility, effectively capturing water movement through the vertical columns of cartilage and newly formed metaphyseal bone.^53–56^ Delgado et al investigated role of DTI in assessing physeal abnormalities in children with high-risk neuroblastoma as compared to controls and found that DTI parameters were lower and associated with short stature in cases as compared to controls.^57^ Jaramillo et al found that tract volumes in physes are more effective predictors of height velocity and total height gain than bone age-based models.^58^

### Intervertebral disc

The nucleus pulposus in the intervertebral discs, a gel-like structure at the centre, primarily functions to distribute hydraulic pressure across the disc during load-bearing activities. The annulus fibrosus, in contrast, is a ring-shaped, multilayered structure composed of concentric lamellae of connective tissue with aligned collagen fibres. During the natural ageing process, the nucleus pulposus gradually undergoes fibrosis and loses proteoglycans, which appear as signal loss on T2-weighted MRI sequences. Furthermore, degradation of the collagen architecture within the annulus fibrosus can compromise disc integrity, potentially leading to bulging of the nucleus pulposus beyond the normal disc perimeter, resulting in herniation.

DWI and DTI are effective non-invasive methods for assessing intervertebral disc integrity and early degenerative changes. Aydin et al evaluated DWI's effectiveness for lumbar disc hernias, concluding it has high sensitivity and specificity for detecting hernias, sequestration, and extrusions.^59^ Zhang et al found that ADC values decreased with age and disc degeneration.^60^ Wang et al and Cui et al studied T1ρ and T2 mapping and ADC in disc degeneration, discovering positive correlations among these values and negative correlations with Pfirrmann grades and age.^61^^,^^62^ Vadapalli et al identified FA maps as potential microstructure biomarkers for normal and degenerating discs.^63^ Kapoor et al found that T2* and ADC value-based grading was highly accurate for assessing disc degeneration compared to Pfirrmann grades, with reduced ADC and T2* values indicating early degeneration.^64^ Shen et al suggested that ADC, FA, and T2* values can quantitatively reflect disc microstructure, aiding in early degeneration detection.^65^ Stein et al reconstructed the 3D microstructure of annular fibrosus fibres using DTI and fibre tracking.^66^ Wei et al evaluated cervical intervertebral disc degeneration with DTI, finding that FA and MD values effectively assess degeneration levels.^67^ Chen et al explored the correlation between DTI parameters and biochemical composition in degenerative discs, indicating that DTI can assess biochemical changes, enhancing clinical diagnosis and treatment evaluation in degenerative disc diseases.^68^

### Synovium

Conventional MRI techniques often struggle to detect synovitis effectively, as they cannot clearly distinguish between synovium and effusion. Contrast-enhanced MRI helps in the visualization of the inflamed synovium and remains the gold standard for imaging synovitis. However, gadolinium-based agents may not be suitable for all patients, prompting the need for alternative imaging methods. DWI, DTI, and DKI have emerged as promising non-contrast MRI techniques for evaluating synovitis. Several studies have explored DWI's role in paediatric patients to avoid gadolinium use.^69–72^ Agarwal et al were the first to investigate DTI for delineating synovitis and they found that while DTI metrics can identify synovial inflammation, they are not superior to conventional MRI for detection and therapeutic response assessment.^73^ Sandford et al aimed to quantify synovitis intensity in osteoarthritis using DTI as an alternative to contrast, finding that quantitative measures can complement morphological assessments to evaluate OA severity and enhance clinical assessments.^74^ Liu et al examined DKI's role in noninvasively identifying synovitis in hand arthritis, suggesting that DKI may be feasible for diagnosing and grading synovitis.^75^

### Peripheral nerves

Diffusion-based sequences like DWI and DTI are gaining recognition for their effectiveness in peripheral nerve imaging, offering important insights into nerve pathology. DWI visualizes water diffusion within tissues, aiding in differentiating between healthy and diseased nerves. This technique improves the detection of nerve entrapments, inflammation, and tumours by highlighting restricted diffusion areas, often linked to pathological changes. MRI with DWI is the gold standard for long-term monitoring of Neurofibromatosis 1-associated tumours due to its excellent soft tissue contrast. Beyond assessing tumour morphology and volume, DWI is valuable for detecting transformations into atypical or malignant stages.^76^ Additionally, DTI is useful for evaluating peripheral nerves in multifocal motor neuropathy and in diabetic peripheral neuropathy.^77^^,^^78^

## DWI in bone tumours

**Figure 4.**
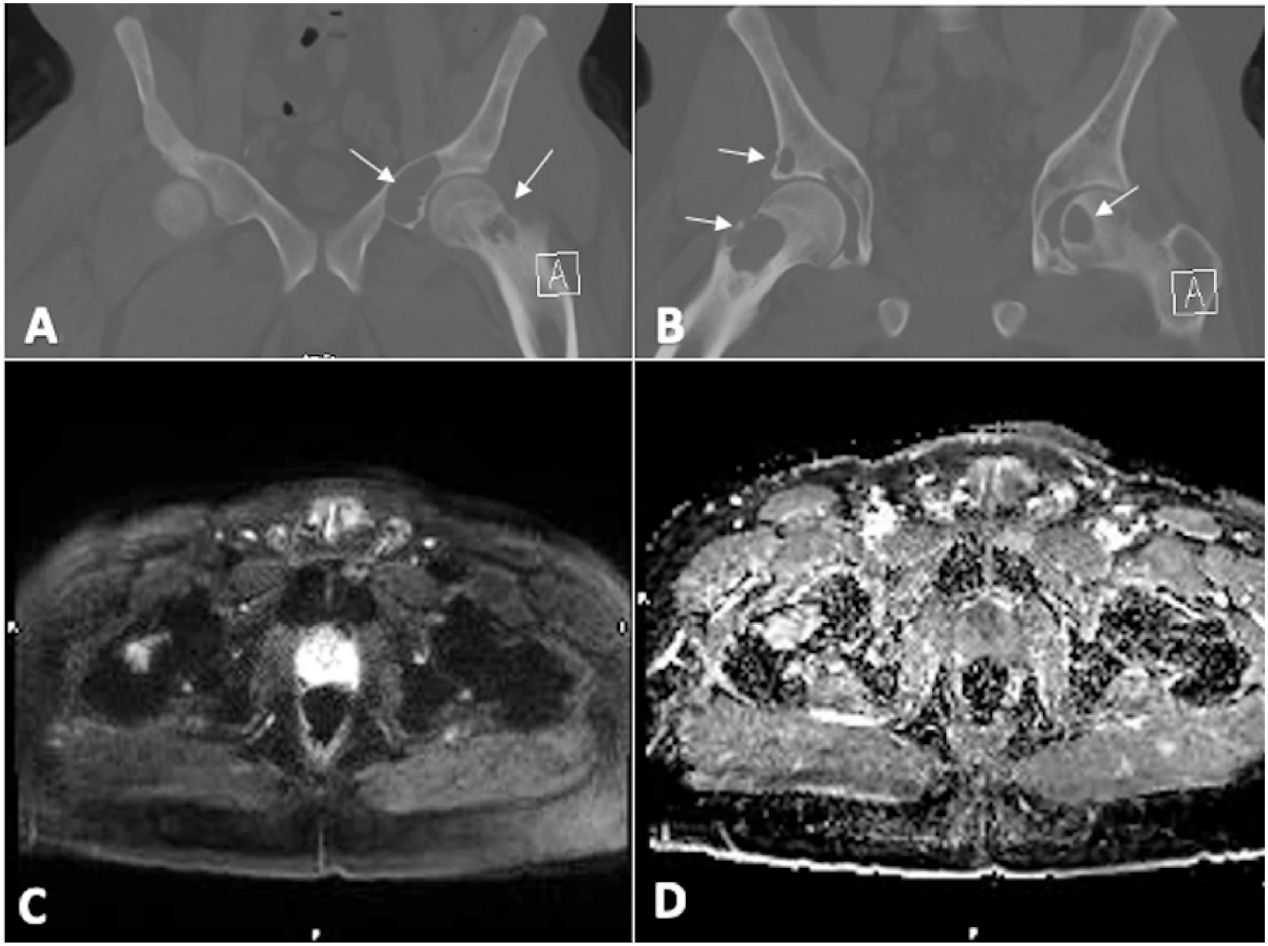
A 51-year-old male with a history of end-stage renal disease who underwent kidney transplant in 2021 presents with low-grade right hip pain. CT images (A and B) demonstrate multiple osteolytic lesions in the pelvic bones and bilateral femur(arrows). The lesion in the right femoral neck exhibited facilitated diffusion (C) with an ADC value of 1.7 (D). Histopathological analysis confirmed it as a brown tumour.

**Figure 5.**
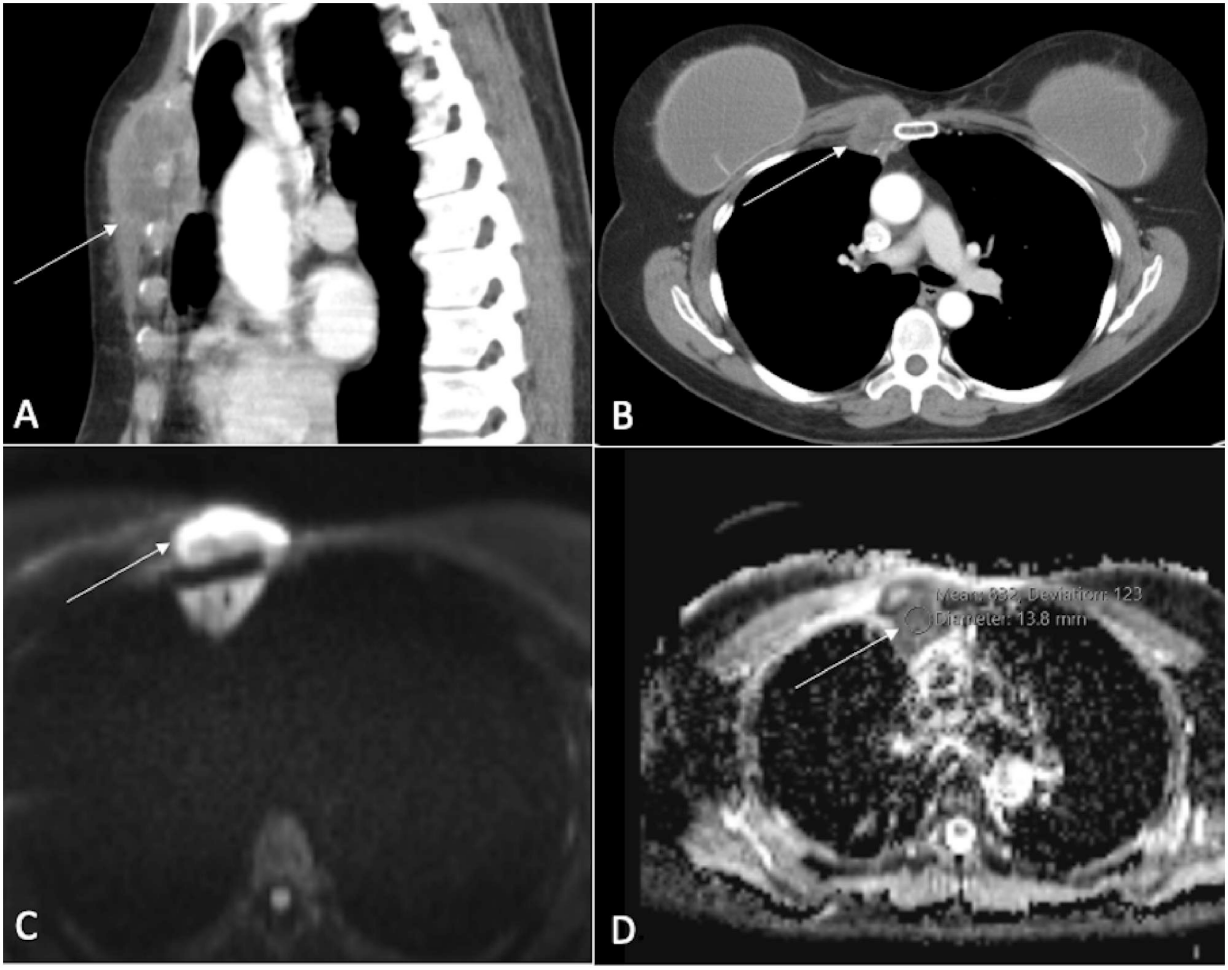
A 72-year-old woman with a breast implant presented with a right sternal mass (arrows) that had been present for 3 months. The lesion appeared heterogeneous on CT images (A and B) and exhibited restricted diffusion on DWI (C), accompanied by a corresponding defect on the ADC map (D). The mean ADC for this case was 0.8. Histopathological examination confirmed the diagnosis of diffuse large B-cell lymphoma.

**Figure 6.**
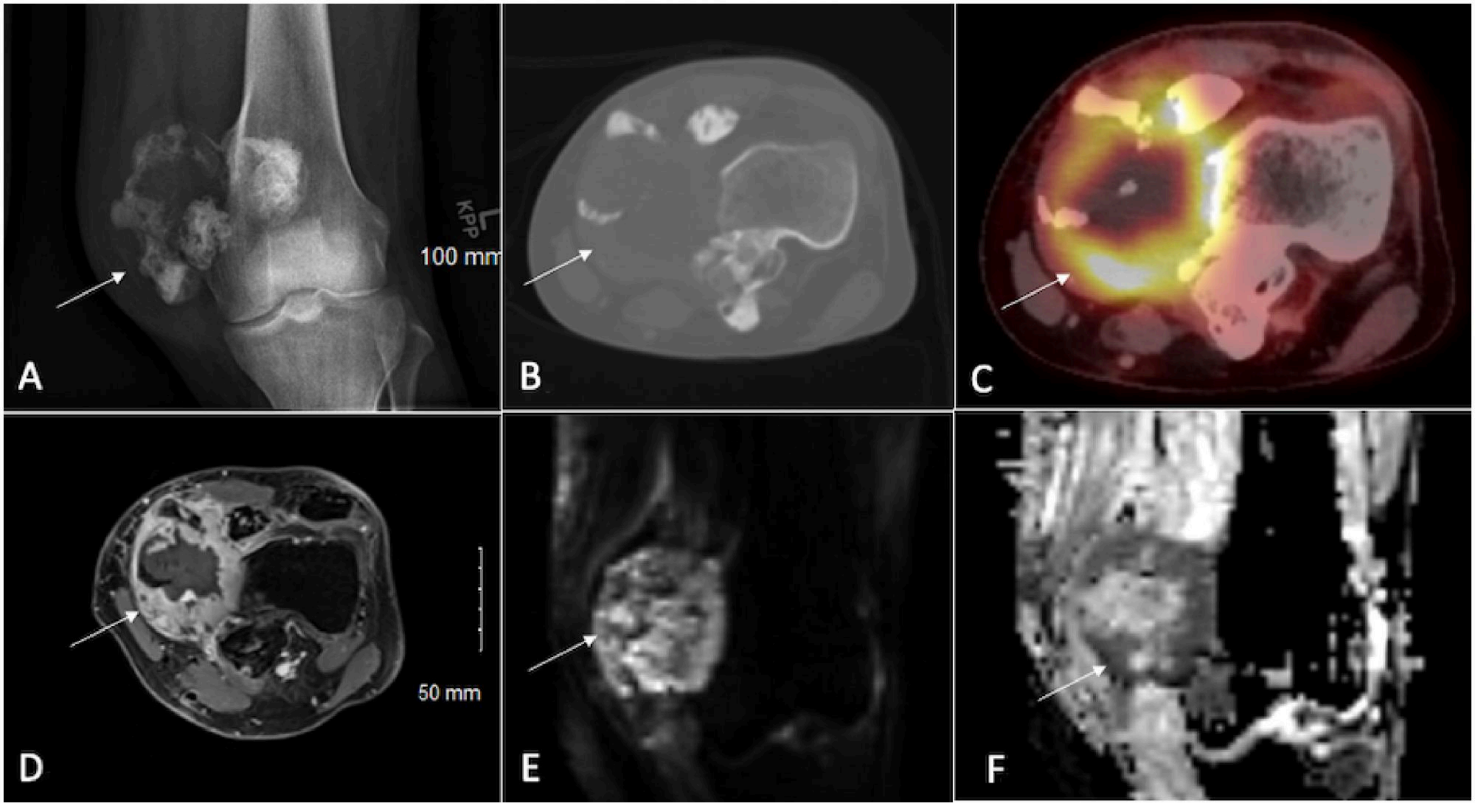
A 30-year-old male presents with increased leg pain and swelling for the past 4 months. Lesions is marked by arrow. Radiograph (A) reveals a large mass lesion on the medial side of the knee, accompanied by a confluent area of calcifications. A CT axial section shows an osteolytic lesion in the medial femoral condyle and adjacent metaphysis, also with calcified regions (B). Positron emission tomography indicates hypermetabolic areas within the lesion (C). A contrast-enhanced MRI (D) displays a heterogeneous enhancement pattern in the lesion’s periphery, alongside a central necrotic area. DWI (E) reveals hyperintensity with a corresponding defect on the ADC map (F), suggesting restricted diffusion. Histopathological examination confirmed a diagnosis of small cell osteosarcoma.

**Figure 7.**
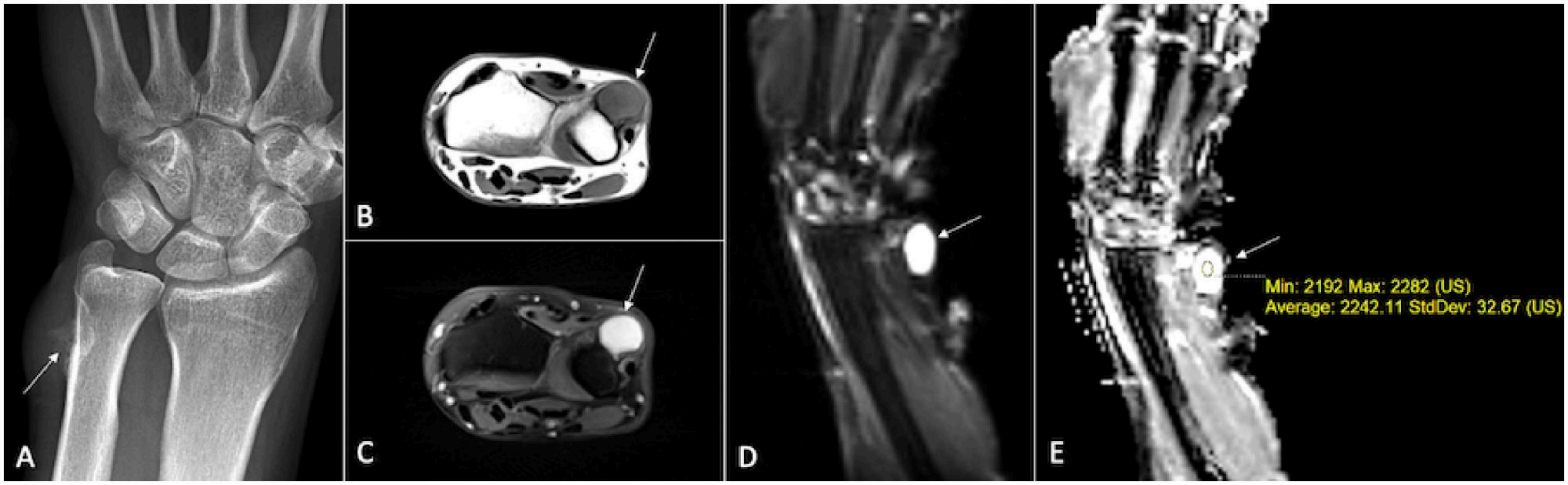
A 47-year-old male presents for evaluation of left wrist pain and swelling that has persisted for 3 years. He reports an increase in size and worsening pain recently. Lesions is marked by arrow. Radiograph reveal periosteal lesion (A) that appeared hypointense on T1-weighted (B) and hyperintense on T2-weighted sequences (C). The lesion showed facilitated diffusion with hyperintensity on DWI (D) and high ADC value (E). Histopathology revealed periosteal chondrosarcoma.

DWI can significantly enhance conventional MRI for suspected bone tumours by improving the visibility of bone lesions through excellent background suppression. It helps differentiate benign lesions, which appear less hyperintense on DWI with high ADC values (Figure 4), from malignant bone lesions that are more hyperintense with low ADC values (Figures 5 and 6).^79^ This distinction aids in quantifying abnormalities, as low ADC values are often seen in high-grade neoplastic processes. In contrast, benign lesions, such as bone cysts, ganglia, and vascular malformations, often display T2-shine-through effects. Using DWI can sometimes eliminate the need for intravenous contrast, reducing imaging costs. High ADC values generally indicate benign lesions (cut-off of 1.15 × 10^−3^ mm^2^/s); however, DWI is less effective for tumours with chondroid and myxoid components, as a substantial reduction in ADC values is generally not apparent in these tissues (Figure 7). Therefore, DWI is not very useful in characterizing cartilaginous tumours. Another exception is giant cell tumours of bone, which exhibit low DWI signal intensity as well as ADC values due to high levels of internal hemosiderin.^2^^,^^80^ DWI utility in bone tumour assessment also suffers from various pitfalls like susceptibility artefacts due to bone, air, and metal (eg, implants, biopsy clips) that can cause magnetic field inhomogeneities, leading to geometric distortion and signal loss; T2 shine-through effect, where high T2 signal (eg, oedema, cystic/necrotic areas) can mimic restricted diffusion; low signal-to-noise ratio (SNR) in densely sclerotic bone tumours (eg, osteoblastic metastases, osteosarcoma); partial volume effects in small lesions; and, variability in ADC measurements due to ROI placement (heterogeneous tumours may give different ADC values depending on sampled area). Currently, DWI is not used for characterizing or staging primary bone tumours, but it can help identify the most cellular tumour components for biopsy.^81^

The potential use of whole-body DWI for metastatic workup is also being explored. Routine assessment of skeletal metastases in high-grade bone sarcomas typically relies on 99 m-Tc MDP whole-body scintigraphy, which has a lower sensitivity for detecting metastatic bone marrow infiltration. Distinguishing metastatic infiltrates from red marrow hyperplasia is challenging. Whole-body MRI with diffusion sequences may help address this issue.^82–84^ Recent studies highlight DWI's value in evaluating post-therapeutic responses in high-grade bone sarcomas. Conventional MRI struggles to assess osteosarcoma response to neoadjuvant chemotherapy, as tumours may not shrink and can appear larger due to surrounding tissue changes. Successful treatment is indicated by over 90% tumour necrosis on histology, which DWI may quantitatively assess through rising mean ADC values. Significant differences in ADC values have been observed between treatment responders (higher ADC values) and non-responders, underscoring DWI's utility in distinguishing post-therapeutic outcomes despite similar tumour volumes.^85–87^

## DWI in soft tissue tumours

**Figure 8.**
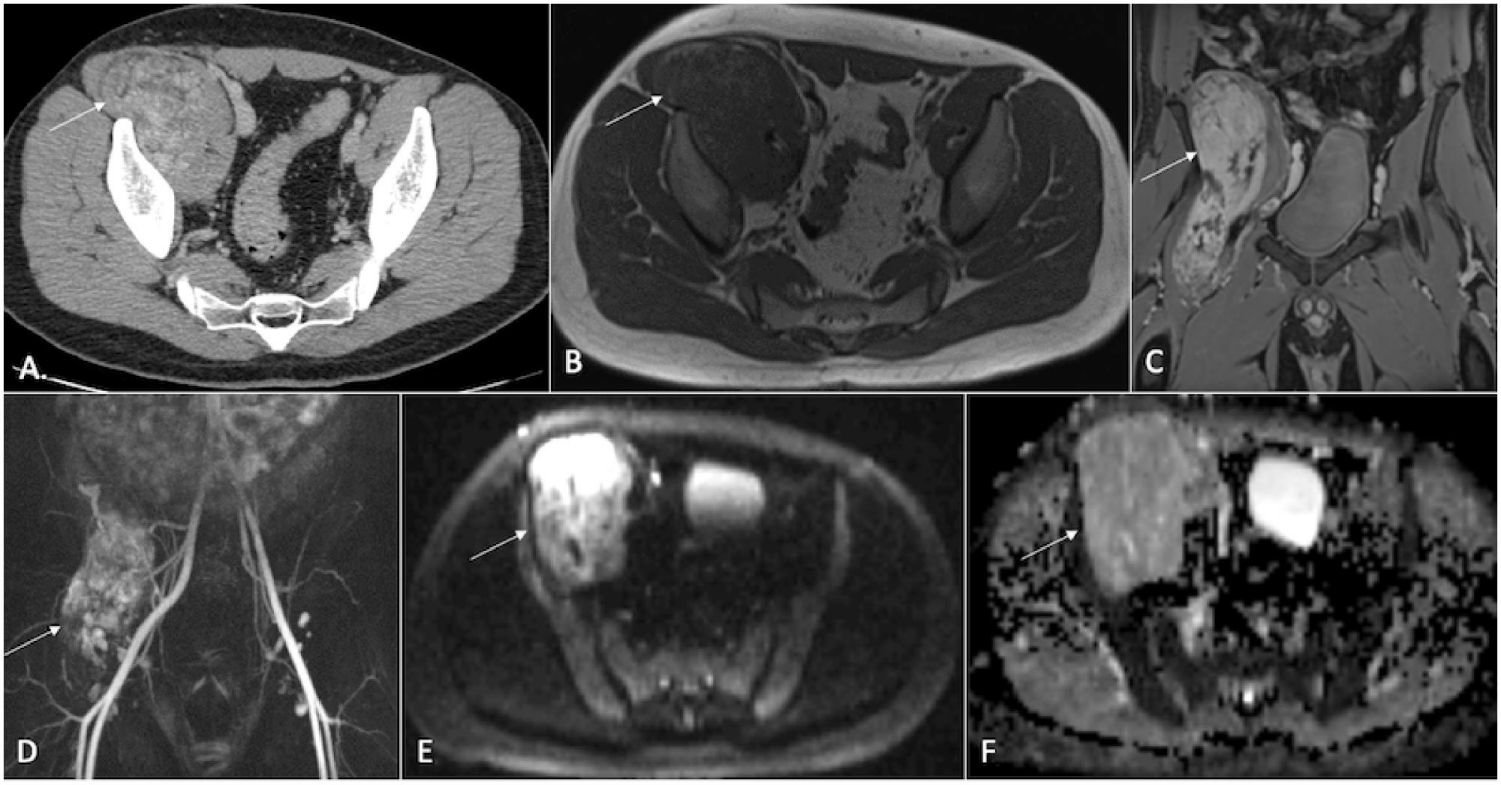
A 27-year-old male has an intramuscular hemangioma located in the right iliopsoas muscle. Lesions is marked by arrow. A post-contrast CT axial section reveals heterogeneous enhancement within the lesion along with fatty streaks. The T1-weighted image displays a hypointense mass with hyperintense streaks suggestive of fat. The post-contrast T1-weighted sequence shows heterogeneous enhancement. The TWIST (Time-resolved Imaging with Stochastic Trajectories) (D) indicates hypervascularity. DWI (E) demonstrates hyperintensity with no corresponding defect on the ADC map (F), suggesting a benign nature.

**Figure 9.**
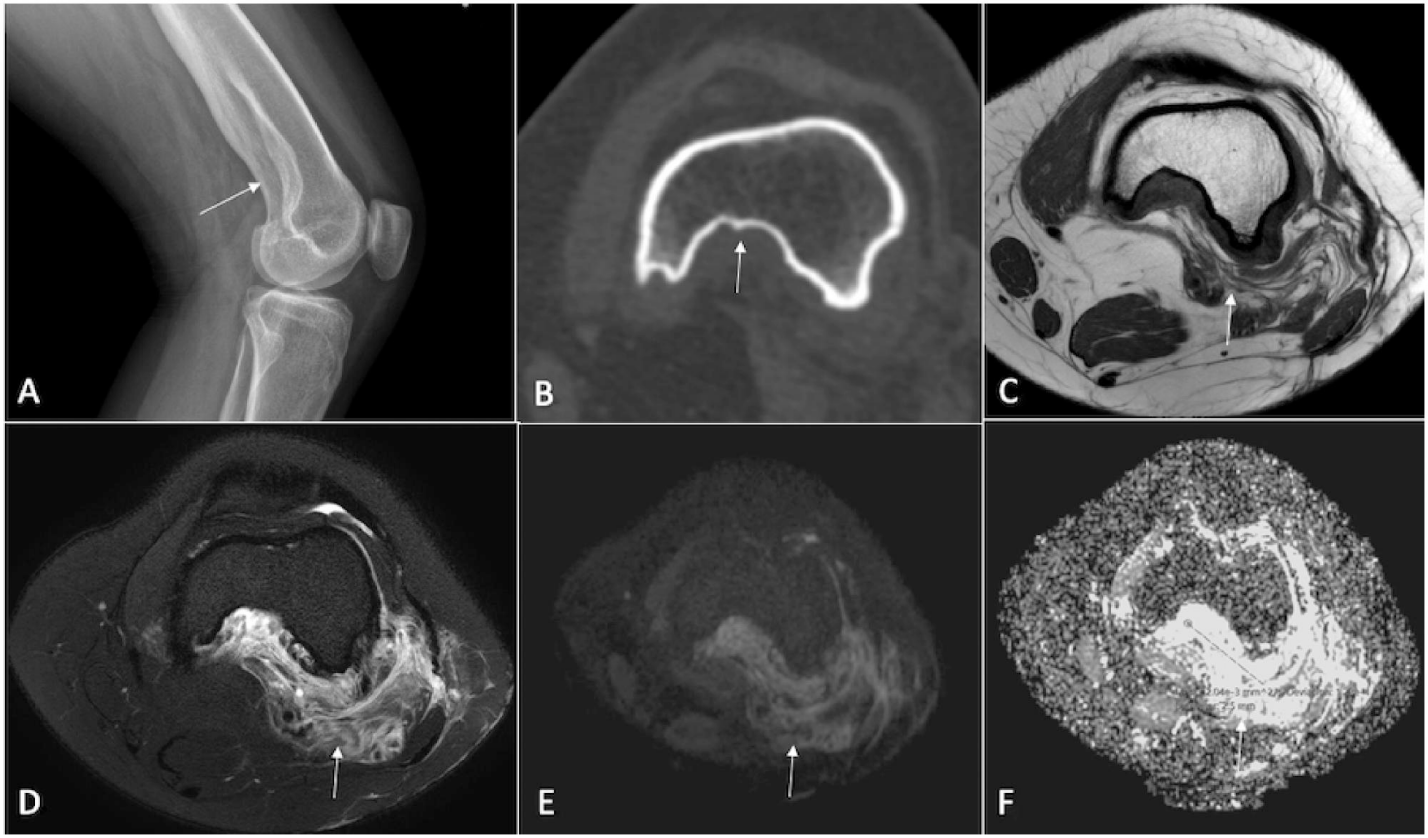
An 18-year-old female with a history of neurofibromatosis type 1 (NF1) presents with deep, stabbing pain in her left lower extremity, radiating from the knee to the foot, along with weakness. Radiograph (A) reveals scalloping (arrow) of the posterior femoral cortex. A CT axial section shows smooth scalloping in the posterior femoral cortex (arrow). The axial T1-weighted image displays a heterogeneously hypointense mass (arrow) with areas of fat. The axial T2-weighted fat-saturated sequence shows a heterogeneously hyperintense mass (arrow) with fat suppression. DWI (E) indicates that the mass (arrow) is hyperintense with facilitated diffusion, displaying high ADC values (arrow) (F). The lesion was diagnosed as neurofibroma.

**Figure 10.**
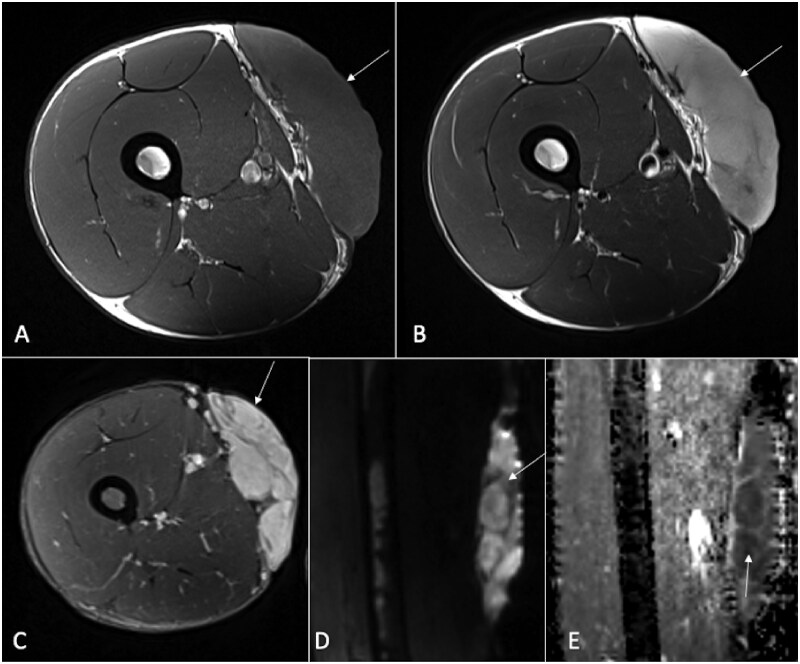
A 31-year-old male presents with a mass in the right thigh (arrow). The axial T1-weighted sequence (A) reveals that the lesion is located in the subcutaneous layer, affecting the skin, and displays uniformly hypointense signals. On the T2-weighted sequence (B), the lesion appears hyperintense, and it demonstrates heterogeneous enhancement on the post-contrast T1-weighted sequence (C). The DWI shows the lesion as hyperintense (D), with a corresponding low signal on the ADC map (E), indicating restricted diffusion. Histopathological examination confirmed the lesion as malignant dermatofibrosarcoma protuberans.

**Figure 11.**
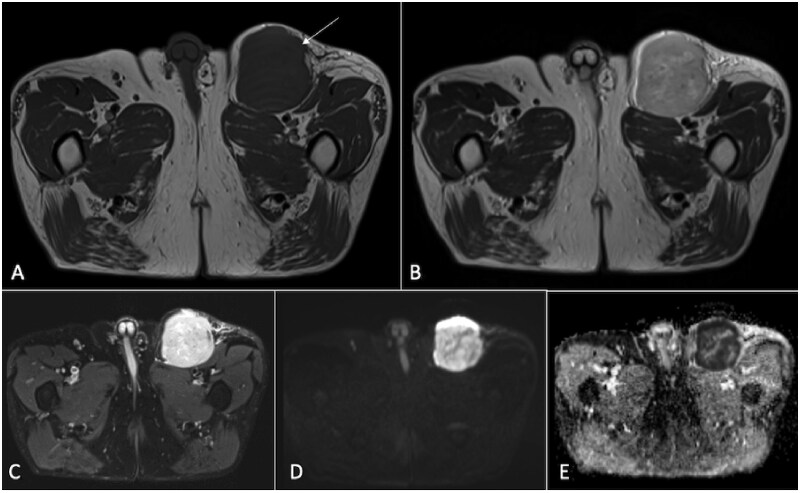
A 63-year-old male presents with a painless, progressively enlarging mass in the left groin (arrow). Imaging reveals a well-defined lesion in the left groin affecting the subcutaneous layer and exerting mass effect on the underlying muscles. It shows hypointense signals on the T1-weighted sequence (A), hyperintense signals on the T2-weighted sequence (B), and heterogeneous enhancement on the post-contrast T1-weighted sequence. The DWI indicates hyperintense signals (D), with a corresponding low signal on the ADC map (E), suggesting restricted diffusion. Histopathological analysis confirmed the lesion as metastatic Merkel cell carcinoma.

DWI is crucial in diagnosing and treating soft tissue tumours and tumour-like lesions. Incorporating DWI into evaluations enhances the characterization of soft tissue tumours, as many solid tumours exhibit non-specific conventional MRI signals and enhancement patterns. Firstly, DWI improves lesion visibility by effectively suppressing fat, blood vessels, and background noise. It helps to differentiate solid lesions from cystic and necrotic ones. The cystic and necrotic lesions will appear hyperintense on DWI with increased ADC values. Similar to bone tumours, DWI provides insights into whether a soft tissue lesion is benign or malignant (ADC < 1.1 × 10^−3^ mm^2^/s), and it helps distinguish between low- and high-grade sarcomas^2^^,^^88^^,^^89^ (Figures 8-11).

Additionally, DWI assists in guiding biopsy procedures by targeting areas with high DWI/low ADC signals, indicating higher cellularity that may not be evident with conventional MRI. DWI is also valuable in monitoring tumour response to treatment; treatment-related cellular necrosis results in increased water diffusivity, leading to higher ADC values. Conversely, low ADC values can indicate tumour recurrence. Tumours may increase in size during radiotherapy due to internal haemorrhage or necrosis; however, significant increases in ADC can confirm a successful treatment response.^90^^,^^91^ Despite its advantages, DWI has limitations. Some benign masses, like pigmented villonodular synovitis (PVNS) and granular cell tumours, may show notable diffusion restriction, while myxoid and chondroid malignant tumours can exhibit less restriction, resembling benign lesions. These limitations should be considered when using DWI for diagnosing soft tissue tumours and tumour-like lesions.^92–94^

## DWI in musculoskeletal infections

**Figure 12.**
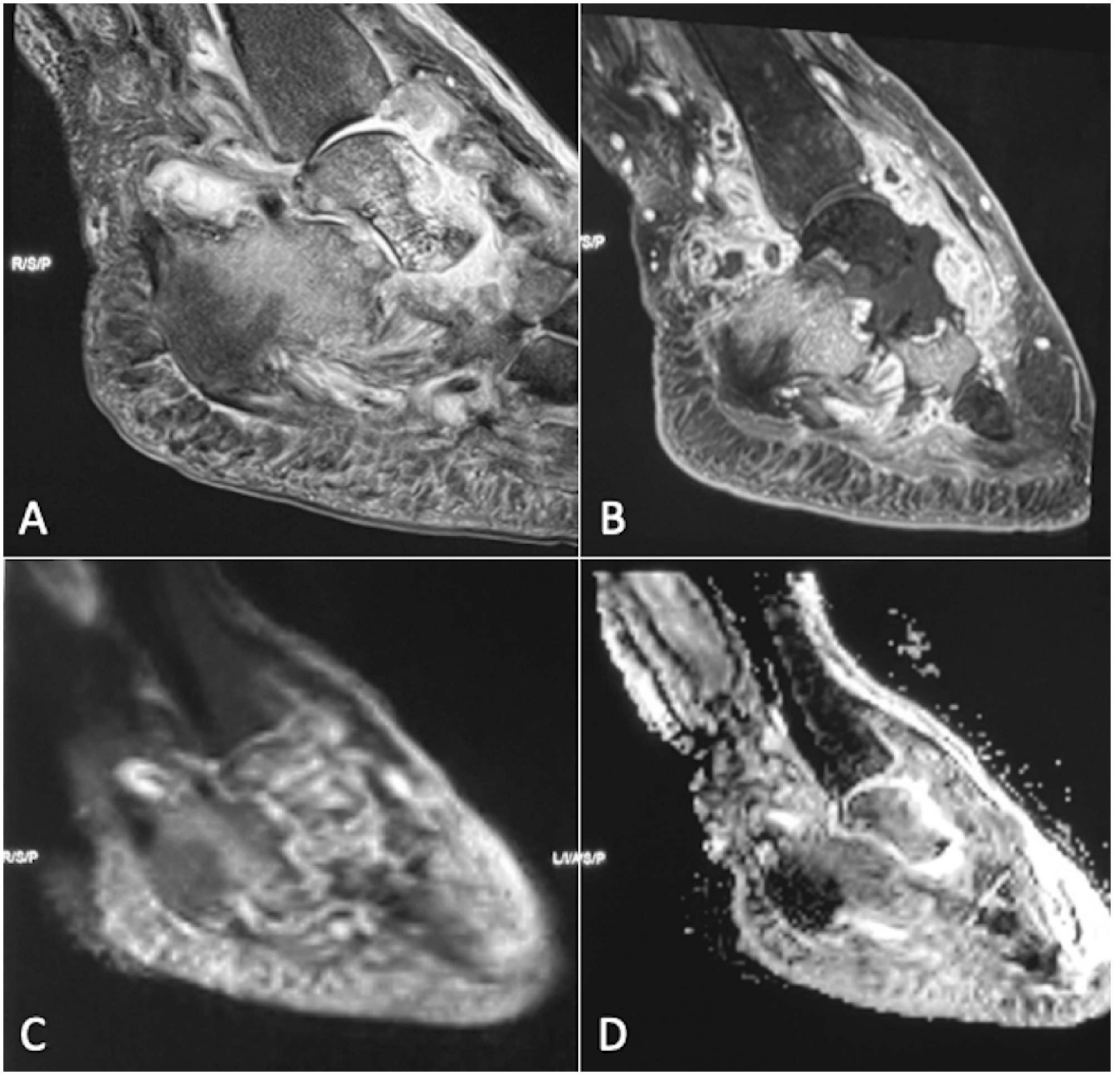
A 54-year-old female presents with a diabetic foot. The PDFS sagittal image (A) reveals involvement of the talus bone and adjacent joints, along with marrow oedema and fluid collections consistent with osteomyelitis. The contrast-enhanced MRI (B) demonstrates enhancement around the periphery of the collection, with non-enhancing necrotic areas within talus with bone erosions of talus, posterior superior calcaneus, cuboid and naviculum. DWI (C) shows hyperintense regions, accompanied by increased signal on the ADC map (D), with ghost sign consistent with proven osteomyelitis. These findings are unlike Charcot neuroarthropathy, which typically shows subchondral bone fractures, disorganization and subchondral cysts.

DWI is highly beneficial for quantifying musculoskeletal infections and is particularly useful for detecting abscesses in areas of devitalized tissue. It helps differentiate cellulitis from simple oedema without needing intravenous contrast, with a cut-off value between 1.2 and 2.0 × 10^−^³ mm^2^/s often cited for differentiating cellulitis from simple oedema.^95^ DWI plays a crucial role in distinguishing between osteomyelitis and neurotrophic joints due to its ability to identify abscesses and infected tissues (Figure 12).^96–98^ The “ghost sign” has been described on T1-weighted sequence and ADC, pointing towards the relative loss of bone within surrounding soft tissue inflammation, which aids in diagnosing osteomyelitis (Figure S1).^99^^,^^100^ Soft tissue oedema and cellulitis are mostly indistinguishable on conventional MRI. However, DWI can aid in this too. Soft tissue oedema appears hyperintense on both DWI and ADC due to facilitated diffusion, whereas cellulitis appears hyperintense on DWI and hypointense on ADC due to underlying inflammation (Figure S2). An abscess, similar to cellulitis, presents as hyperintense on DWI with significantly low ADC values with a cut-off value of 1.098 × 10^−3^ mm^2^/s (Figure S3). Some authors have proposed modifications to the DWI sequence to reduce image distortion and enhance fat saturation, making it a more effective choice for evaluating lower extremity infections.^101^

## DWI in joint pathologies

DWI plays a significant role in imaging arthritis. Normal cartilage has low ADC values due to its structured organization, but when damaged, it will appear bright on DWI with increased ADC values.^49^ Areas affected by “chondromalacia and blistering” display facilitated diffusion, whereas regions of fibrocartilage and chondrocalcinosis show restricted diffusion. DWI is also valuable in differentiating between PVNS and synovial osteochondromatosis. PVNS typically forms tumour-like masses and does not calcify, making it similar to synovial osteochondromatosis on conventional imaging. On DWI and ADC, PVNS shows a low signal due to the “T2 black effect,” while synovial osteochondromatosis presents with a high signal due to the “T2 shine through effect” (Figure S4).^2^ Most intra-articular tumour-like lesions are benign, including ganglion cysts, hemangiomas, and chondromas. However, synovial sarcoma, a high-grade sarcoma, can occasionally occur intra-articularly and display low ADC values. Additionally, DWI is useful for detecting and grading sacroiliitis and enthesitis, providing a more unbiased method to assess and monitor the inflammation during treatment.^102^

## Pitfalls associated with DWI applications in the musculoskeletal system

DWI imaging pitfalls fall into technical and interpretation-related categories.

1. *Susceptibility artefacts*: This is due to magnetic susceptibility differences at tissue interfaces (eg, bone-soft tissue, metal implants, gas) that causes geometric distortions and signal loss common near orthopaedic hardware, fractures, or post-surgical sites. It can be mitigated by using multi-shot EPI to minimize distortions, applying higher bandwidth and parallel imaging and using non-EPI DWI techniques (eg, PROPELLER, BLADE, or RESOLVE).
2. *T2 shine-through effect*: High T2 signal can mimic restricted diffusion, leading to false-positive findings (eg, in cysts, oedema, or fluid-filled lesions). This can be avoided by reviewing ADC maps (true restricted diffusion shows low ADC values) and using exponential DWI (eDWI) or high b-values (≥800 s/mm^2^) to reduce T2 influence.
3. *Low signal-to-noise ratio (SNR) in fibrous/bony tissues*: Muscles, tendons, and cortical bone have inherently low diffusion signal, reducing diagnostic confidence. Optimizing b-values, using higher magnetic strength with appropriate coils can mitigate this.
4. *Motion artefacts (patient & physiological)*: Voluntary (patient movement) and involuntary (cardiac/pulsation, breathing) motion degrade image quality. Mitigation involve using fast DWI sequences (single-shot EPI) or navigator-based motion correction and applying restraints/padding to minimize limb movement and respiratory/ECG gating for physiological motions.
5. *Fat suppression challenges*: Inhomogeneous fat suppression (eg, near bone marrow, metal implants) can obscure pathology. This can be mitigated by using STIR (Short Tau Inversion Recovery) for uniform fat suppression or Dixon-based fat/water separation for better uniformity.
6. *Partial volume effects*: Small lesions near bone or muscle boundaries may be missed due to averaging with adjacent tissues. This can be reduced by using thin slices (≤4 mm) and high-resolution DWI and combining it with anatomical sequences (T1/T2-weighted MRI) for correlation.
7. *B-value selection and standardization issues*: Inconsistent b-values across institutions make ADC quantification non-reproducible. Standardize protocols (eg, *b* = 0, 50, 400, 800 s/mm^2^) should be used and phantom calibration should be performed for ADC reproducibility.
8. *False-negatives in highly cellular tumours*: Some tumours (eg, myeloma, lymphoma) may show high ADC due to necrosis or oedema. DWI should always be interpreted with conventional MRI sequences. Dynamic contrast-enhanced (DCE) MRI or PET/CT for can be used for better characterization.
9. *Anatomically challenging areas*: Optimizing DWI in anatomically challenging regions like the foot requires addressing susceptibility artefacts, distortion, and signal dropouts caused by air-tissue interfaces, bony structures, and complex anatomy. Align the imaging plane along the long axis of the foot (eg, coronal oblique) to minimize geometric distortion. Use high-resolution 3D localizers to ensure proper alignment before DWI acquisition and if possible, use 3D DWI (eg, RESOLVE or reduced-FOV techniques) to allow reformatting in any plane.

## Conclusion

DWI is a valuable tool in musculoskeletal imaging, offering insights into various tissues and conditions. It enhances lesion visibility, differentiates tissue types, and supports diagnoses such as arthritis, synovitis, and bone tumours. Despite its advantages, DWI faces technical pitfalls, including motion artefacts and susceptibility issues, which can be addressed with advanced imaging techniques like parallel imaging and multishot EPI. Interpretation challenges, such as misdiagnosing benign lesions as malignant, underscore the need to integrate DWI with clinical history and conventional MRI. Visual assessment of DWI/ADC in musculoskeletal imaging is useful but not infallible. It is most reliable when integrated with other MRI sequences, used for high contrast clear-cut scenarios (eg, abscess, highly cellular tumours) and interpreted by experienced radiologists aware of pitfalls.

For nuanced diagnoses (eg, low-grade vs. high-grade tumours), quantitative ADC analysis improves accuracy. By using DWI as a supplemental technique and not in isolation, we can improve diagnostic accuracy and patient outcomes in musculoskeletal assessments.

## Supplementary Material

tzaf019_Supplementary_Data
