# Supplementary figures and images for: Diffusion weighted imaging in musculoskeletal system: where are we now?
Source: BJR Open. 2025 Jul 22;7(1):tzaf019. doi: 10.1093/bjro/tzaf019 (PMC12342750; doi:10.1093/bjro/tzaf019)

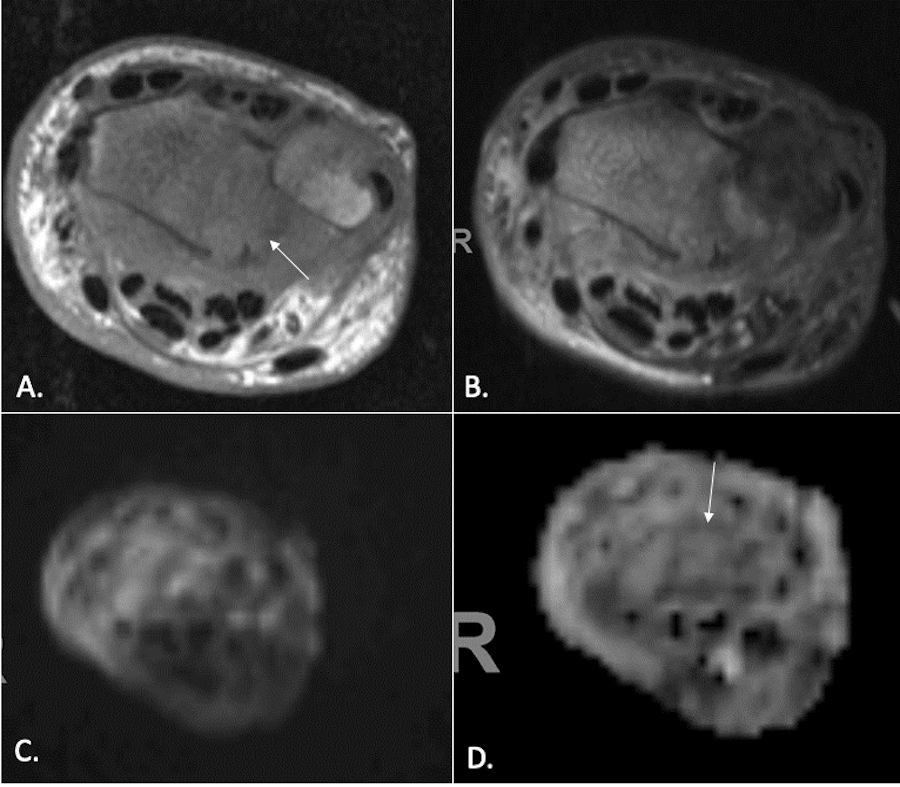

Supplement: tzaf019_Supplementary_Data [file tzaf019_supplementary_data.zip › Suppl 1.tiff]

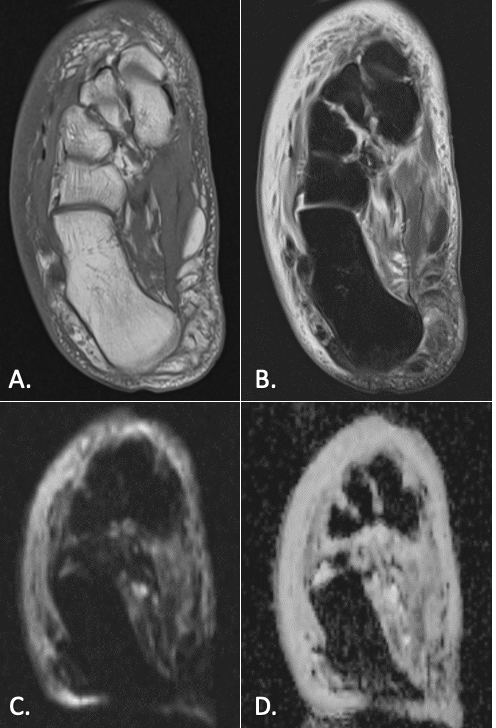

Supplement: tzaf019_Supplementary_Data [file tzaf019_supplementary_data.zip › Suppl 2.tiff]

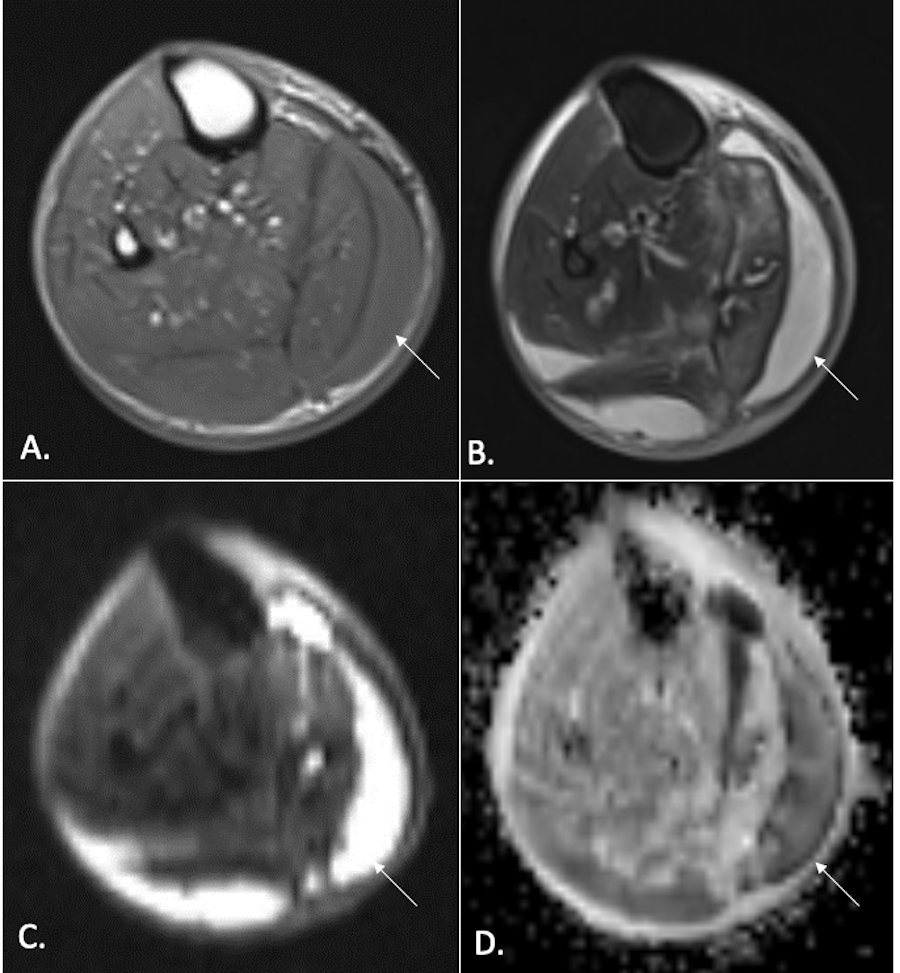

Supplement: tzaf019_Supplementary_Data [file tzaf019_supplementary_data.zip › Suppl 3.tiff]

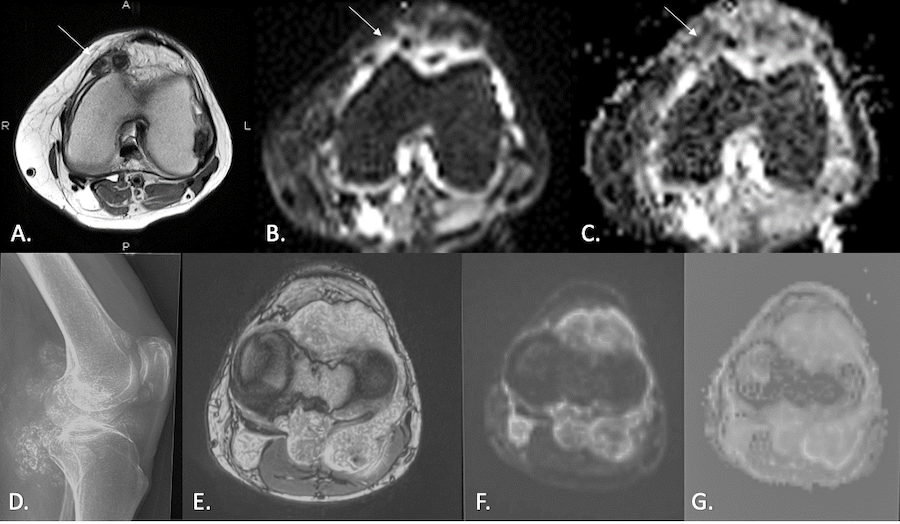

Supplement: tzaf019_Supplementary_Data [file tzaf019_supplementary_data.zip › Suppl 4.tiff]
